# Nonfatal Firearm Injuries by Intent in the United States: 2016-2018 Hospital Discharge Records from the Healthcare Cost and Utilization Project

**DOI:** 10.5811/westjem.2021.3.51925

**Published:** 2021-05-21

**Authors:** Kathryn Schnippel, Sarah Burd-Sharps, Ted R. Miller, Bruce A. Lawrence, David I. Swedler

**Affiliations:** * Everytown for Gun Safety Support Fund, New York, New York; † Pacific Institute for Research and Evaluation, Calverton, Maryland

## Abstract

**Introduction:**

In addition to the nearly 40,000 firearm deaths each year, nonfatal firearm injuries represent a significant public health burden to communities in the United States. We aimed to describe the incidence and rates of nonfatal firearm injuries.

**Methods:**

We calculated nonfatal firearm injury estimates using the Healthcare Cost and Utilization Project of the Agency for Healthcare Research and Quality, including the Nationwide Emergency Department Samples and the National Inpatient Samples. We used the International Classification of Diseases, 10th Revision, Clinical Modification to identify firearm injury episodes. Deaths in the emergency department (ED) or as inpatients were excluded.

**Results:**

In addition to the 118,171 persons shot and killed by firearms from 2016–2018, 228,380 people were shot (ratio 1.9:1) and treated at a hospital ED or admitted to hospital, a rate of 23.4 nonfatal firearm injury episodes per 100,000 population. The number of nonfatal injury episodes varied by year: 2018 had the lowest at 69,692, compared to 84,776 in 2017 and 73,912 in 2016. Unintentional injury episodes were the most frequent, accounting for 58.5% (n = 81,217) and 38.9% (n = 34,820) of total nonfatal firearm hospital discharges from the ED and inpatients, respectively. Assault episodes were the next most frequent, at 36.3% (n = 50,482) of ED and 49.5% (n = 44,290) of inpatient discharges. The highest rate of nonfatal firearm injury by five-year age group was for 20- to 24-year-olds. With an annual rate of 73.53 per 100,000 population, the rates for ages 20–24 were more than 10 times higher than the rates for patients younger than 15 or 60 years and older. More than half (53.4%, n = 121,884) of hospital-treated, nonfatal firearm injury episodes were patients living in ZIP codes with a median household income in the lowest quartile, compared to 7.5% (n = 17,102) for patients residing in the highest income quartile ZIP codes, a sevenfold difference.

**Conclusion:**

For every person shot and killed by a gun in the US, two more are wounded. Unlike firearm deaths, which are predominantly suicides, most nonfatal firearm injury episodes are unintentional or with an assault intent. Having a reliable source of nonfatal injury data is essential to understanding the incidence of firearm injuries.

## INTRODUCTION

The story of gun violence in the United States is often told through the deaths that are reported through the National Vital Statistics System by the US Centers for Disease Control and Prevention (CDC): more than 100 gun deaths each day.^1^ But an often-overlooked part of today’s gun violence crisis are nonfatal injuries. Understanding the contours of these injuries—where, to whom, and how often—is essential for developing solutions. Knowing more about nonfatal gun injuries is essential information to enable doctors, emergency medical technicians, police departments, policymakers, and trauma hospitals to plan for future need. It is also important for studying the survival rate of those wounded by a gunshot and could provide important signals for understanding trends in the criminal use of firearms. In an effort to fill this critical gap, we analyzed hospital administrative data from the Healthcare Cost and Utilization Project (HCUP) for 2016–2018, the most recent years available at the time the research was undertaken, using data on emergency department (ED) and inpatient hospital discharges for nonfatal firearm injuries.

## METHODS

Nonfatal firearm injury incidence estimates are calculated from HCUP databases. Coordinated by the Agency for Healthcare Research and Quality, HCUP databases bring together the data collection efforts of state data organizations, hospital associations, and private data organizations, the HCUP Data Partners. The Partners are listed on the HCUP-US website at https://www.hcup-us.ahrq.gov/db/hcupdatapartners.jsp. Emergency department discharges are from the Nationwide Emergency Department Sample s (NEDS) for 2016–2018.^2^ Inpatient (admitted) discharges are from the corresponding National Inpatient Sample (NIS).^3^ We applied discharge-level weights to the survey sample in NEDS and NIS to calculate representative estimates for the US.

Unweighted, a single year of NEDS includes approximately 33.5 million hospital discharges that started in the ED; the weighted sample sums to 145 million ED discharges. For 2018, NEDS approximated a 20% stratified sample of hospital-owned EDs in the US and included data from 990 hospitals across 36 partnering states and the District of Columbia.^2^ Unweighted, a single year of NIS includes approximately seven million inpatient hospital admissions to community hospitals, excluding rehabilitation and long-term acute care hospitals; the weighted sample provides estimates for more than 35 million admissions. For 2018, NIS approximated a 20% random sample of discharges from each hospital in the 47 partnering states and the District of Columbia.^3^

To avoid double-counting across the two datasets, we dropped inpatient admissions to the same hospital and transfers to other inpatient facilities from the NEDS dataset as it was assumed the hospital admission would be represented by the NIS dataset. To avoid double-counting fatal injuries reported by the CDC, we excluded firearm-related hospital discharges that resulted in death in the ED or as an inpatient. Additionally, as NEDS and NIS are both cross-sectional snapshots, we did not count subsequent encounters or sequelae.

### Analysis

We extracted hospital discharge records for patients with firearm-related injuries using the National Center for Health Statistics’ *International Classification of Diseases*, *10^th^ Revision, Clinical Modification* (ICD-10) codes^4^ for initial encounters related to firearm discharges. We excluded injuries as a result of firearm malfunction or injuries of any intent from gas, air, or spring-operated guns, paintball guns, and rubber bullets. For the 2016 dataset, ICD-10 codes related to injuries were captured under a specific variable for external cause of morbidity. Beginning with the 2017 dataset, ICD-10 codes for external causes are included in the diagnosis codes^2^,^3^; firearm injuries were extracted from all possible diagnoses (e.g., up to 35 unique codes in NEDS), regardless of other diagnoses reported.

**Population Health Research Capsule**

What do we already know about this issue?

*Fatal and nonfatal firearm injuries represent a significant public health burden to the US; however, there is little data on nonfatal injuries.*

What was the research question?

*We examined hospital discharges to understand which patients and communities are most impacted by nonfatal gun injuries.*

What was the major finding of the study?

*For each firearm death, there are two injuries; nearly all firearm injuries are unintentional or with an assault intent.*

How does this improve population health?

*Prevention efforts must address the disproportionate burden of nonfatal firearm injuries on racial minorities and low-income and urban communities.*

Hospital discharges were assumed to represent an injury episode. One person may have sustained multiple gunshot wounds in the same firearm injury episode and would be counted once for the hospital discharge. It is also possible that one person may have multiple firearm injury episodes in a year and, therefore, the incidence of firearm injury episodes may be higher than the number of unique persons experiencing a firearm injury episode in the year.

The ICD-10 codes are categorized according to injury intent: assault (including assault by terrorism); self-harm (including attempted suicide); legal intervention (shootings by police); injuries considered unintentional; and injuries where the intent was undetermined. The larger ICD-10 external injury category for legal intervention includes operations of war and military operations; however, the counts reported here are only for legal intervention involving firearm discharge where the law enforcement officer, bystander, or suspect was injured. A full list of included codes is available in the supplemental appendix.

The ICD-10 codes Y90–Y99 are available for supplementary factors related to external injuries. Evidence of alcohol involvement, place of occurrence (e.g., residence, school, business, public space), and activity at time of injury were explored. However, as most firearm injuries had no supplementary factor codes or no information provided in these codes (e.g., coded as unspecified or not applicable), we did not report these supplementary factors.

We provide descriptive statistics using variables as available and coded in the datasets. Injuries were described using injury intent and whether the patient was discharged from the ED or after inpatient admission. For inpatients, the NIS files also include information on the “All Patients Refined Diagnosis Related Groups” (APR DRG) subclassifications for the risk of mortality (minor, moderate, major or extreme likelihood of dying) and the severity of illness (minor, moderate, major, or extreme loss of function).^3^ Patient individual characteristics were described for gender (male or female) and age from both NEDS and NIS. Combined race and ethnicity (categorized as Asian or Pacific Islander, Black, Hispanic, Native American, White, and other races including mixed race) was available for inpatients only.

Both datasets included the urban-rural classification of the county of patient residence, categorized as the following: large central metro (counties with significant population of a metropolitan statistical area of one million or more); large fringe metro (counties in a metropolitan statistical area but not considered central); medium metro (counties in a metropolitan statistical area of 250,000 to 999,999 population); small metro (counties in a metropolitan statistical area of less than 250,000 population); and micropolitan or noncore (rural).^5^ Both datasets also included the median household income quartile of the patient ZIP code as categorized in the dataset; the quartiles were defined for each year, with the lowest quartile including ZIP codes with a median income of up to $42,999 in 2016 and $45,999 in 2018. Hospital characteristics common to both datasets were limited to the US Census region (Midwest, Northeast, South, and West).

We calculated annual crude population rates per 100,000 population on the weighted national estimates using the population file from HCUP released in 2020 for the three years 2016–2018.^6^ All analysis was done in Stata, release 16 (StataCorp., College Station, TX) using the survey commands to account for the weighting.

The HCUP datasets are public use files that do not include any patient-level identifying information; therefore, this was not considered human subject research. Counts less than 10 are suppressed as per restrictions on the dataset. The study is presented in accordance with STROBE reporting guidelines for cross-sectional observational studies.^7^

## RESULTS

From 2016–2018, hospitals provided an estimated 228,380 episodes of care (95% confidence interval [CI], 213,824 to 242,936) for nonfatal shootings in the United States, a rate of 23.40 per 100,000 population (95% CI, 21.91 to 24.89). Excluding follow-up visits, national estimates include 138,935 (60.8%) nonfatal firearm injury episodes treated only in the ED (95% CI, 125,737 to 152,133) and 89,445 (39.2%) treated as inpatients (95% CI, 83,386 to 95,504).

From the NEDS alone across the three years, there were 63,150 initial encounter episodes where firearm was the mechanism of injury (a weighted national estimate of 264,886) in total for all injury intents. To avoid double-counting NIS admissions, we excluded 26,197 (41.5% of firearm-coded injuries) NEDS discharges to an inpatient admission to the same hospital or transferred as inpatient (weighted estimate of 109,432). Additionally, we excluded from the analyses 3840 patients who died in the ED (10.4%, weighted estimate n = 16,419) and 1,651 patients who died in hospital (8.5%, weighted estimate n = 8,255). The combined weighted estimate of 24,674 deaths excluded represent 85% of the CDC-reported 29,009 firearm injury deaths in medical facilities as inpatient, outpatient or ED, and dead on arrival.

### Injury Characteristics

**Figure 1:**
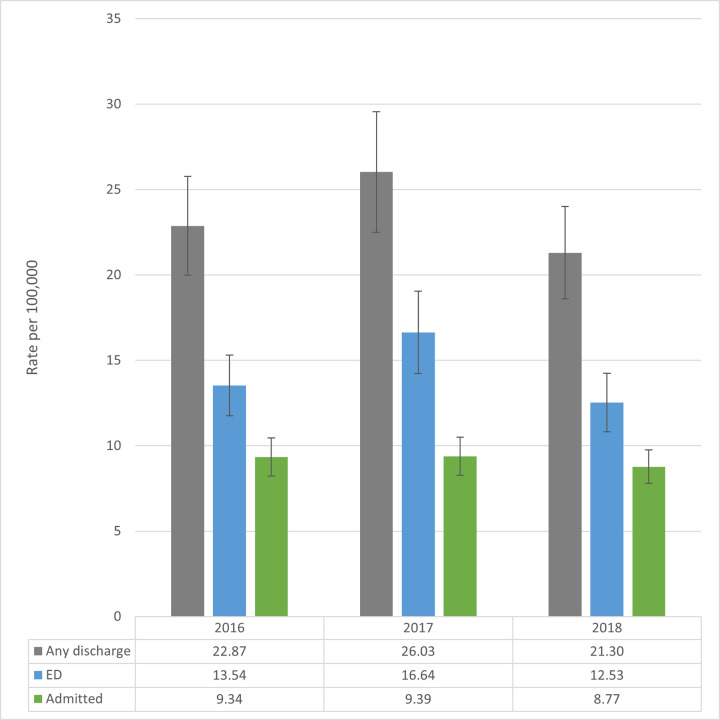
Rate of nonfatal firearm injury episodes in the United States per 100,000 population, by year, 2016–2018. Admitted nonfatal firearm injury episodes from the National Inpatient Sample (2016–18). Emergency department (ED) nonfatal firearm injury episodes from the Nationwide Emergency Department Sample (2016–18). Population from Healthcare Cost and Utilization Project files. Lines represent 95% confidence intervals for weighted survey estimates.

There is no clear trend in the rate of nonfatal firearm injury episodes (Figure 1) over these three years, and confidence intervals overlap. The number of nonfatal firearm injury episodes varied by year: 2018 had the lowest incidence of 69,692, compared to 84,776 in 2017 and 73,912 in 2016. The difference was driven by ED episodes in NEDS. The 2018 incidence of 40,992 episodes was 24.4% lower than the 54,206 in 2017. For inpatient episodes, the NIS national estimate of 28,700 episodes was 6.1% lower in 2018 compared to 2017 (n = 30,570). On average, there were 76,127 nonfatal firearm injury episodes per year, including 46,312 discharged from the ED and 29,815 treated as inpatients.

**Figure 2:**
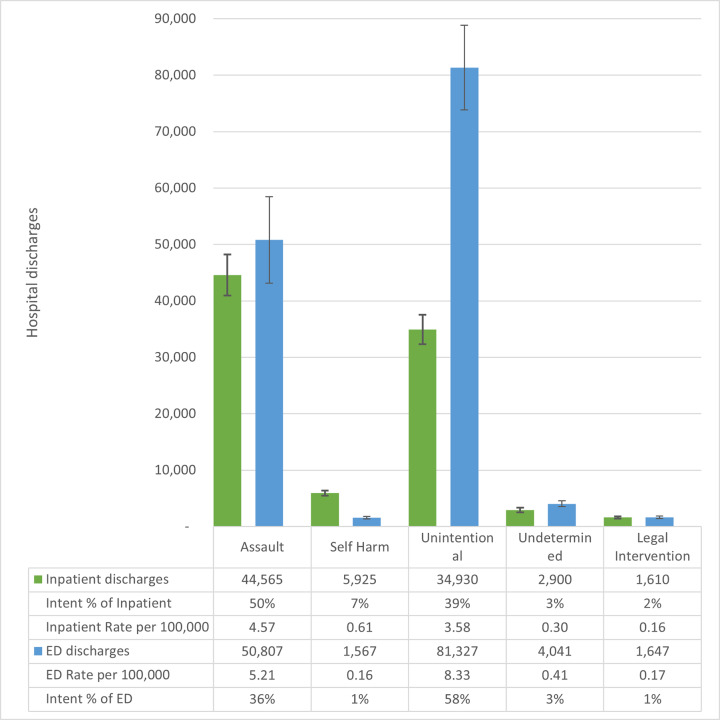
Number, rate per 100,000 population, and proportion of admitted and emergency department (ED) discharges for nonfatal firearm injuries in the United States, by injury intent, 2016–2018. Admitted firearm injury episodes estimated from the National Inpatient Sample (2016–18). Emergency department discharges estimated from the Nationwide Emergency Department Sample (2016–18). Lines represent 95% confidence intervals for weighted survey estimates. Proportions are for rows and may not total to 100% because of rounding.

Across both ED and inpatient episodes, firearm type was usually categorized as other or unspecified (68.9%, n = 157,316). Among the injuries with firearm type categorized, across all intents, injuries were most frequently attributed to handguns (80.2%) compared to long guns (e.g., rifles or shotguns, 19.8%). Overall, unintentional injury episodes were the most frequent, accounting for 58.5% (n = 81,217) and 38.9% (n = 34,820) of total nonfatal firearm hospital episodes from the ED and inpatients, respectively (Figure 2). Assault episodes were the most frequent among inpatient discharges (49.5%, n = 44,290) and second highest for ED discharges (36.3%, n = 50,482). The other three intents combined – intentional self-harm, undetermined and legal intervention – made up 5.2% (n = 7,236) and 11.6% (n = 10,335) of ED and inpatient episodes, respectively.

The NIS dataset also includes variables about the risk of mortality and severity of the injury using the APR DRG subclassifications for risk of mortality and severity of illness as detailed in Table 1. Among survivors of hospital-admitted injury, 12.2% (n = 10,900) had been categorized as being at extreme risk of dying based on the firearm injuries sustained. A higher number of patients, 18,770 (21.0% of the nonfatal firearm inpatient discharges) experienced an injury severe enough to be categorized as causing extreme loss of function. Specific to intent, nonfatal self-harm injury patients had the highest frequency of being classified as extreme risk of dying (20.6%, n = 1,220) or causing an extreme loss of function (29.6%, n = 1,755), and unintentional injury patients had the lowest relative frequency for both severity classifications (10.3%, n = 3,895 and 17.1%, n = 6,475).

The mean length of inpatient hospital stay was 7.95 days (95% CI, 7.73 to 8.16). Routine discharge from inpatient admission was the most frequent outcome (74.2%, n = 66,370 patients). However, 12.4% (n = 11,060) were discharged to an “other” facility such as skilled nursing or intermediate care, and 8.5% (n = 7,625) were discharged to home health care.

### Patient Characteristics

**Figure 3:**
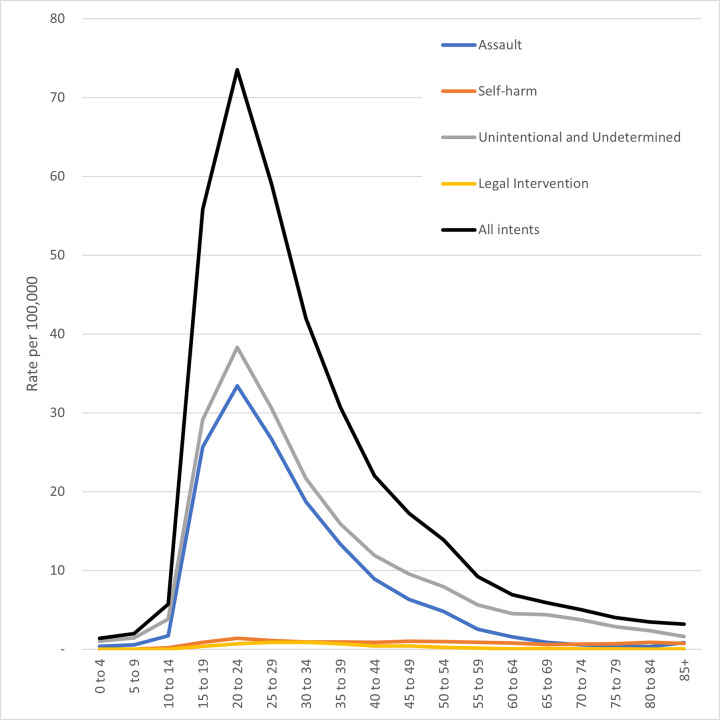
Rate of nonfatal firearm injury episodes in the United States per 100,000 population, by 5-year age group and Injury Intent, 2016–2018. Inpatient hospital discharges from the National Inpatient Sample and emergency department discharges from the Nationwide Emergency Department Sample. Population from Healthcare Cost and Utilization Project files.

Most (87.3%, n = 199,320) nonfatal firearm injury episodes from 2016–2018 were among men and boys, a rate of 41.47 episodes per 100,000 population (95% CI, 38.83 to 44.12) (Figure 3). Two-thirds of patients (67.0%, n = 153,115) seen in hospital for firearm injuries were between the ages of 15–34 years. By far, the highest rate by five-year age group was for 20- to 24-year-olds with a rate of 73.53 per 100,000 population (95% CI, 67.86 to 79.20), more than 10 times higher than the rates for patients younger than 15 or 60 years and older. Nearly 1 in 10 (9.2%, n = 20,921) nonfatal firearm injury hospital episodes were pediatric patients under 18 years old (95% CI, 19,451 to 22,392).

**Figure 4:**
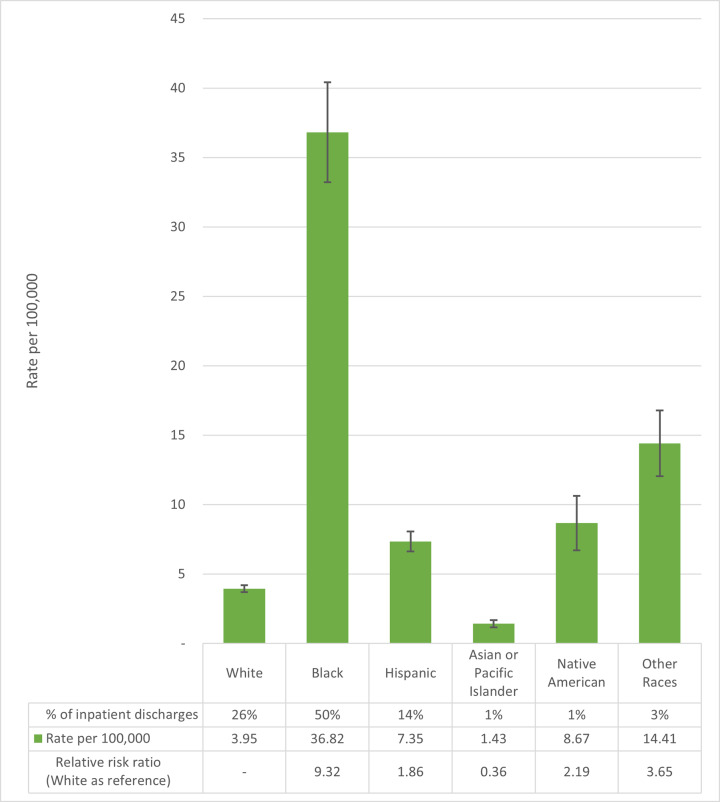
Row proportion, rate per 100,000 population, and relative risk of nonfatal firearm injury episodes requiring inpatient admission for the United States, by combined race and Hispanic origin, 2016–2018, calculated from inpatient discharges in the National Inpatient Sample. Lines represent 95% confidence intervals for weighted survey estimates.

Combined patient race and ethnicity were only available for the inpatient data (Figure 4). Black people, with 50.1% of the nonfatal firearm injury episodes requiring inpatient admission (n = 44,835) and a rate of 36.82 inpatient episodes per 100,000 people (95% CI, 33.22 to 40.41), have the highest rate, more than nine times higher than white people (3.95 per 100,000, 95% CI, 3.71 to 4.19).

### Community and Hospital Characteristics

Across all intents, rates of nonfatal firearm injury episodes were similar with overlapping confidence intervals in counties categorized as small metros, micropolitan, and noncore rural communities, on average 21.84 per 100,000 (Table 2). However, rates were approximately twice as high in counties with large central metro areas at 31.48 per 100,000 (95% CI, 27.93 to 35.03) compared to the suburban surrounding counties (large fringe metros, 14.40 per 100,000). Patients living in ZIP codes with a median household income in the lowest quartile (ranging from less $43,000 per year in 2016 to less than $46,000 per year in 2018) accounted for 53.4% (n = 121,884, 95% CI, 111,629 to 132,138) of all nonfatal firearm injury episodes compared to 7.5% (n = 17,102, 95% CI, 15,728 to 18,475) for patients residing in ZIP codes with median household incomes at the highest income quartile (at $71,000 to $79,000 and above), a sevenfold difference.

There were also considerable differences among the four US Census regions (Northeast, Midwest, South, and West). Half (50.0%, n = 114,224) of nonfatal firearm injury episodes occurred in the 16 states of the American South, with a rate of 30.81 per 100,000 (95% CI, 27.97 to 33.65), approximately twice that of the Northeast and of the West (13.22 and 16.85 per 100,000, respectively).

## DISCUSSION

In addition to the 118,171 persons shot and killed by firearms from 2016–2018,^1^ an estimated 228,380 people survived their injuries. Compared to firearm deaths, nearly two times as many people were shot and treated at a hospital ED and/or admitted to hospital, a rate of 23.40 nonfatal firearm injury episodes per 100,000 population. The socioeconomic and demographic makeup of those injured by firearms each year, with an average of 208 people per day who are wounded with a firearm and survive, tells an important story for focusing prevention efforts. As is evident from this analysis, the overall distribution of gun injuries by both demographic group and income is extremely uneven.

Eighty seven percent of those who visit a hospital for a gunshot wound are male. The age group most impacted by nonfatal firearm injuries is young adults 20–24 years old, with a rate that is over 10 times higher than both youth (under 15) and older adults (≥60). The rate of nonfatal firearm injury episodes requiring inpatient admission for Black people is over nine times higher than that of white people and the highest rate of the major US racial and ethnic groups. The Latino rate of nonfatal gun injuries is double that of non-Latino white people. These overlaps put Black young adult males at very high risk of nonfatal gunshot injuries. Through the lens of income, more than half of all nonfatal firearm injury episodes affect residents of communities in the poorest quartile for household incomes.

The distribution of those who are treated and survive also varies significantly by intent, with implications for prevention efforts that can reduce morbidity and mortality. The rate of those who are shot and survive in core central cities is triple the rate in rural areas and small towns. The burden of nonfatal firearm injury, particularly from assault and unintentional injuries, seems to be similar to firearm homicide in its disproportionate impact on Black adolescent boys and young men in urban communities.^1^

This analysis of the nonfatal firearm injury episodes from both ED and inpatient discharges provides a unique and more complete picture of the incidence of firearm injuries. Previous analyses of HCUP data often focused on either NEDS^8^,^9^ or NIS^10^,^11^ but not both. A study of NEDS from 2006–2014 found the incidence of nonfatal ED visits (including those admitted as inpatients) to be 23.2 per 100,000, similar to the rate of 23.40 found here for 2016–2018.^12^ A study of trends over time from the NEDS and NIS data was outside the scope of this descriptive analysis of the incidence of nonfatal firearm injury episodes. The three years included here had wide variation, particularly the 24% decrease from 2017 to 2018 in ED discharges for patients who survived and were not admitted as inpatients. A NIS-specific study found that the number of injuries was increasing over time from 1993–2014; further, the total of 24,445 inpatient admissions for firearm injuries in 2014 was lower than the average 29,815 estimated here for 2016– 2018.^10^ Our estimates, however, are 26% lower than the estimated 58,912 nonfatal admissions and 43,440 ED cases in 1992, similar to the decline in non-suicide firearm deaths over the same period (19,607 in 1992 and average of 15,649 for 2016– 2018).^13^,^14^

The average annual incidence of 76,127 nonfatal firearm injuries for 2016–2018 is far lower than the 2016 estimate of 110,968 reported by the CDC in its Firearm Injury Surveillance Study.^15^ The CDC has not reported nonfatal firearm injuries on its Web-based Injury Statistics Query and Reporting System website for 2016–2019 because the coefficient of variation exceeds 30% and the CDC has determined national weighted estimates are therefore unreliable.^16^ The limitations of CDC firearm injury data stem in part from its small sample size of EDs. Currently, the agency’s survey includes only about 66 hospitals—less than 2% of all hospitals in the US.^15^ In comparison, hospitals included in the NIS cover 97% of the US population; therefore, the HCUP estimates are likely more accurate than CDC nonfatal injury reports.^17^

Of note, the NEDS dataset includes patients who were either admitted to the same hospital or discharged from the ED to another inpatient facility. The NIS sample of discharges from inpatient hospitals is both weighted to represent inpatient admissions and includes more states than the NEDS sample (weighted to represent hospitals with ED facilities) and therefore was assumed to be more accurate. While these NEDS inpatient estimates were excluded from this joint analysis of the NEDS and NIS, the differences in using estimates from only NEDS are important to consider. Across the three years, on average the NEDS estimate of inpatient admissions was 14.1% higher than the NIS estimate (n = 102,039 vs n = 89,445). However, most of this difference was in 2017. The 2017 estimate from NEDS of nonfatal firearm injuries requiring hospital admission was 35.6% higher than the NIS estimate for 2017 (n = 41,438 vs n = 30,570). In contrast, the NEDS weighted estimate for nonfatal inpatient admissions was 3.7% and 2.1% higher in 2016 and 2018, respectively.

One possible explanation for the 2017 outlier is that one or more of the trauma centers responding to the 2017 Las Vegas mass shooting where over 400 were treated for gunshot wounds from a single event^18^,^19^ was included in the NEDS sample of hospital EDs.^2^ The weighting from the NIS sample of discharges, rather than hospitals, would not have been impacted to the same degree. In light of the differences in national weighted estimates from the choice of NEDS or NIS datasets, the switch from ICD-9 to ICD-10 coding that occurred mid-2015, and the short-term variations described here, further research to ensure that differences over time are not the result of methodology is needed before we can draw conclusions from data patterns.

The tens of thousands of Americans injured by firearms each year face many difficulties, including severe injury and hospitalization with its associated medical bills. The cost of nonfatal firearm injuries in 2013 has been estimated at $2.5 billion for the medical treatment alone, with an additional $23.5 billion for mental healthcare, police and criminal justice response, lost wages, and lost quality of life.^20^

The physical disability and costs of rehabilitation continue when discharged from the hospital. Analyses of the 2013– 2014 HCUP Nationwide Readmissions Database found that 7.6% of patients hospitalized with nonfatal firearm injuries are readmitted within 30 days, and that patients with firearm injuries were more likely to be readmitted within 90 days following discharge compared to patients injured as either pedestrians or occupants in a motor vehicle collision.^21^,^22^ On average, 9.5% of the cost of hospitalizations for nonfatal firearm injuries is due to readmission within the first six months of injury.^23^ The trauma experienced can also have lasting impact for survivors of nonfatal gun injuries, even for those whose physical wounds heal completely. A follow-up survey of patients discharged from hospital with a gunshot wound found that, years after being shot, respondents had lower reported measures of mental health, physical health, emotional support, and ability to participate in social roles. There were worse scores for patients with regard to alcohol use and substance abuse, and patients were more likely to screen positive for post-traumatic stress disorder.^24^

Importantly, hospital-based violence interventions programs have been shown to be cost saving while reducing the risk of further violent injury.^25^ Similarly, interventions such as Counseling on Access to Lethal Means (CALM) have been successfully implemented in ED settings and may be an important tool in reducing the risk of firearm suicide.^26^ The importance of lethal means counseling and reducing access to guns for persons at risk of suicide is clear from this analysis of nonfatal firearm injuries. Nearly two-thirds of gun deaths each year are by suicide, with the remaining one-third from homicide, yet intentional self-harm accounts for only 3% of the nonfatal firearm hospital discharges each year. The small number of persons seen as inpatients (5925) and even smaller number seen and discharged from the ED (1567) for nonfatal, intentional self-harm firearm injuries compared to the 71,224 firearm suicides for 2016–2018 points to the high lethality of firearms as a means for suicide.^1^,^27^

## LIMITATIONS

While NEDS and NIS are the largest and most representative samples of hospitalizations in the US, they are both just samples and not a full census of hospitalizations. As the differences between NEDS and NIS estimates of inpatient admissions and the differences between HCUP and CDC datasets show, included or excluded hospitals and communities can create a large difference in national estimates. This analysis attempted to look across NEDS and NIS by dropping NEDS patients who had an outcome of inpatient admission or transfer to inpatient hospital and assuming that these patients were represented in NIS. If these patients were incorrectly coded in NIS as being subsequent rather than initial visits, the counts presented here would underestimate the burden of injury.

As with other analyses of external causes of injury, the ICD-10 codes may not accurately reflect the intent because of limited information at the time of the hospital encounter. In cases where the intent of a shooting injury is unclear, and in the absence of affirmative documentation on the incident, unintentional injuries may be overestimated and intentional self-harm and assault injuries may be underestimated.^28^ NEDS and NIS also are both surveys of hospitalizations and exclude nonfatal firearm injuries that may have been managed in clinicians’ offices or urgent care facilities separate from hospitals and therefore likely underestimate less severe injuries from firearms.

## CONCLUSION

There is a persistent and urgent need to understand nonfatal firearm injury episodes seen in EDs and as inpatients in hospitals across the United States. Nonfatal firearm injury episodes on average occur at a rate twice that of firearm deaths. This descriptive analysis points to large disparities in terms of the high rate and heavy burden of nonfatal firearm injury episodes particularly in low-income, urban communities and among Black adolescent boys and young men. Policies and interventions to reduce gun violence must focus on the most impacted communities and prioritize community- and evidence-based solutions that address these disparities.

## Supplementary Information



## Figures and Tables

**Table 1:** Number, row proportion, and rate per 100,000 population by injury characteristics for inpatient nonfatal firearm injury episodes for the United States, 2016–2018, based on inpatient hospital discharges from the National Inpatient Sample.

Category	Description	Total	Row proportion	Crude rate per 100,000
Total inpatient	Inpatient admission	89,445	36.1%	9.16
APR DRG risk of mortality	Minor likelihood of dying	50,655	56.6%	5.19
	Moderate likelihood of dying	14,110	15.8%	1.45
	Major likelihood of dying	13,755	15.4%	1.41
	Extreme likelihood of dying	10,900	12.2%	1.12
APR DRG severity of injury	Minor loss of function	17,170	19.2%	1.76
	Moderate loss of function	30,540	34.1%	3.13
	Major loss of function	22,940	25.6%	2.35
	Extreme loss of function	18,770	21.0%	1.92
Disposition of patient	Routine	66,370	74.2%	6.80
	Transfer to short-term hospital	2,295	2.6%	0.24
	Transfer other, includes skilled nursing	11,060	12.4%	1.13
	Home health care	7,625	8.5%	0.78
	Against medical advice	1,875	2.1%	0.19

*APR DRG*, All Patients Refined Diagnosis Related Groups.

**Table 2:** Number, row proportion, and rate per 100,000 population by community characteristics for nonfatal firearm injury episodes for the United States, 2016–2018, based on emergency department discharges from the Nationwide Emergency Department Sample and inpatient hospital discharges from the National Inpatient Sample. Population from Healthcare Cost and Utilization Project files.

Category	Description	Total	Row proportion	Crude rate per 100,000
Hospital census region	Northeast	22,306	9.8%	13.22
	Midwest	52,736	23.1%	25.80
	South	114,224	50.0%	30.81
	West	39,113	17.1%	16.85
Patient residence urbanization	Large central metro	95,303	41.7%	31.48
	Large fringe metro	34,913	15.3%	14.40
	Medium metro	46,352	20.3%	22.72
	Small metro	19,547	8.6%	21.95
	Micropolitan	17,329	7.6%	21.05
	Noncore	12,657	5.5%	22.52
Patient ZIP median household income	Quartile 1 (lowest)	121,884	53.4%	50.04
	Quartile 2	50,737	22.2%	20.87
	Quartile 3	33,679	14.7%	13.68
	Quartile 4 (highest)	17,102	7.5%	7.09
