# Supplementary material for: Nonfatal Firearm Injuries by Intent in the United States: 2016-2018 Hospital Discharge Records from the Healthcare Cost and Utilization Project
Source: West J Emerg Med. 2021 May 21;22(3):462–70. doi: 10.5811/westjem.2021.3.51925 (PMC8203029; doi:10.5811/westjem.2021.3.51925)
Supplement: Supplementary file 1 [file wjem-22-462-s001.docx]

### Supplemental Exhibit 1. ICD-10-CM Codes included in analysis

Assault

X93.XXXA Assault by handgun discharge, initial encounter

X94.0XXA Assault by shotgun, initial encounter

X94.1XXA Assault by hunting rifle, initial encounter

X94.2XXA Assault by machine gun, initial encounter

X94.8XXA Assault by other larger firearm discharge, initial encounter

X94.9XXA Assault by unspecified larger firearm discharge, initial encounter

X95.8XXA Assault by other firearm discharge, initial encounter

X95.9XXA Assault by unspecified firearm discharge, initial encounter

Terrorism

Y38.4X1A Terrorism involving firearms, public safety official injured, initial encounter

Y38.4X2A Terrorism involving firearms, civilian injured, initial encounter

Y38.4X3A Terrorism involving firearms, terrorist injured, initial encounter

Intentional self-harm

X72.XXXA Intentional self-harm by handgun discharge, initial encounter

X73.0XXA Intentional self-harm by shotgun discharge, initial encounter

X73.1XXA Intentional self-harm by hunting rifle discharge, initial encounter

X73.2XXA Intentional self-harm by machine gun discharge, initial encounter

X73.8XXA Intentional self-harm by other larger firearm discharge, initial encounter

X73.9XXA Intentional self-harm by unspecified larger firearm discharge, initial encounter

X74.8XXA Intentional self-harm by other firearm discharge, initial encounter

X74.9XXA Intentional self-harm by unspecified firearm discharge, initial encounter

Unintentional

W32.0XXA Accidental handgun discharge, initial encounter

W33.00XA Accidental discharge of unspecified larger firearm, initial encounter

W33.01XA Accidental discharge of shotgun, initial encounter

W33.02XA Accidental discharge of hunting rifle, initial encounter

W33.03XA Accidental discharge of machine gun, initial encounter

W33.09XA Accidental discharge of other larger firearm, initial encounter

W34.00XA Accidental discharge from unspecifiedfirearms or gun, initial encounter

W34.09XA Accidental discharge from other firearms, initial encounter

Undetermined intent

Y22.XXXA Handgun discharge, undetermined intent, initial encounter

Y23.0XXA Shotgun discharge, undetermined intent, initial encounter

Y23.1XXA Hunting rifle discharge, undetermined intent, initial encounter

Y23.2XXA Military firearm discharge, undetermined intent, initial encounter

Y23.3XXA Machine gun discharge, undetermined intent, initial encounter

Y23.8XXA Other larger firearm discharge, undetermined intent, initial encounter

Y23.9XXA Unspecified larger firearm discharge, undetermined intent, initial encounter

Y24.8XXA Other firearm discharge, undetermined intent, initial encounter

Y24.9XXA Unspecified firearm discharge, undetermined intent, initial encounter

Legal intervention intent

Y35.001A Legal intervention with unspecified firearm discharge, law enforcement official injured initial encounter

Y35.002A Legal intervention with unspecified firearm discharge, bystander injured, initial encounter

Y35.003A Legal intervention with unspecified firearm discharge, suspect injured, initial encounter

Y35.011A Legal intervention with injured by machine gun, law enforcement official injured initial encounter

Y35.012A Legal intervention with injury by machine gun, bystander injured, initial encounter

Y35.013A Legal intervention with injury by machine gun, suspect injured, initial encounter

Y35.019A Legal intervention with injury by machine gun, unspecified person injured, initial encounter

Y35.021A Legal intervention with injury by handgun, law enforcement official injured initial encounter

Y35.022A Legal intervention with injury by handgun, bystander injured, initial encounter

Y35.023A Legal intervention with injury by handgun, suspect injured, initial encounter

Y35.029A Legal intervention with injury by handgun, unspecified person injured, initial encounter

Y35.031A Legal intervention with injury by rifle pellet, law enforcement official injured initial encounter

Y35.032A Legal intervention with injury by rifle pellet, bystander injured, initial encounter

Y35.033A Legal intervention with injury by rifle pellet, suspect injured, initial encounter

Y35.039A Legal intervention with injury by rifle pellet, unspecified person injured initial encounter

Y35.091A Legal intervention with firearm discharge, law enforcement official injured initial encounter

Y35.092A Legal intervention with firearm discharge, bystander injured, initial encounter

Y35.093A Legal intervention with firearm discharge, suspect injured, initial encounter

Y35.099A Legal intervention with firearm discharge, unspecified person injured, initial encounter

### Supplemental Exhibit 2. Nonfatal Firearm Injury Episodes, Injury Characteristics, Rate per 100,000 with 95% Confidence Intervals, 2016 to 2018

| **Category** | **Total (n=228,380)** | **Lower Estimate**  **(n=213,824)** | **Upper Estimate**  **(n=242,936)** |
| --- | --- | --- | --- |
| **Total Nonfatal Firearm Injury Episodes** | 23.40 | 21.91 | 24.89 |
| **Discharge type** |  |  |  |
| NEDS | 14.23 | 12.88 | 15.59 |
| NIS | 9.16 | 8.54 | 9.79 |
| **Admission month** |  |  |  |
| Jan | 21.59 | 20.39 | 23.59 |
| Feb | 19.69 | 16.76 | 19.46 |
| Mar | 19.75 | 18.68 | 21.56 |
| Apr | 21.74 | 19.79 | 23.06 |
| May | 22.70 | 21.28 | 24.97 |
| June | 23.30 | 21.10 | 24.84 |
| Jul | 25.21 | 23.70 | 27.65 |
| Aug | 24.08 | 22.65 | 26.40 |
| Sept | 22.80 | 20.69 | 24.25 |
| Oct | 23.96 | 22.54 | 26.27 |
| Nov | 21.74 | 19.81 | 23.04 |
| Dec | 21.57 | 20.39 | 23.55 |
| **Admission on weekend** |  |  |  |
| Monday-Friday | 21.27 | 20 | 23 |
| Saturday-Sunday | 28.62 | 27 | 30 |
| **Intent** |  |  |  |
| Not firearm |  |  |  |
| Assault | 9.71 | 8.85 | 10.57 |
| Self-harm | 0.76 | 0.71 | 0.81 |
| Unintentional | 11.89 | 11.08 | 12.70 |
| Undetermined | 0.71 | 0.64 | 0.78 |
| Legal enforcement | 0.33 | 0.30 | 0.36 |
| **APR DRG Severity of Injury (Inpatient only)** |  |  |  |
| Minor loss of function | 1.76 | 1.64 | 1.88 |
| Moderate loss of function | 3.13 | 2.92 | 3.34 |
| Major loss of function | 2.35 | 2.17 | 2.53 |
| Extreme loss of function | 1.92 | 1.77 | 2.07 |
| **APR DRG Risk of Mortality (Inpatient only)** |  |  |  |
| Minor risk of dying | 5.19 | 4.84 | 5.54 |
| Moderate risk of dying | 1.45 | 1.33 | 1.56 |
| Major risk of dying | 1.41 | 1.29 | 1.53 |
| Extreme risk of dying | 1.12 | 1.03 | 1.21 |

### Supplemental Exhibit 3. Nonfatal Firearm Injury Episodes, Community Characteristics, Rate per 100,000 with 95% Confidence Intervals, 2016 to 2018

| **Category** | **Total (n=228,380)** | **Lower Estimate**  **(n=213,824)** | **Upper Estimate**  **(n=242,936)** |
| --- | --- | --- | --- |
| **Total Nonfatal Firearm Injury Episodes** | 23.40 | 21.91 | 24.89 |
| **Hospital Census Region** |  |  |  |
| Northeast | 13.22 | 10.34 | 16.09 |
| Midwest | 25.80 | 22.02 | 29.58 |
| South | 30.81 | 27.97 | 33.65 |
| West | 16.85 | 15.13 | 18.58 |
| **Patient Residence Urbanization Classification** |  |  |  |
| Large central metro | 31.48 | 27.93 | 35.03 |
| Large fringe metro | 14.40 | 12.70 | 16.09 |
| Medium metro | 22.72 | 20.62 | 24.82 |
| Small metro | 21.95 | 19.01 | 24.88 |
| Micropolitan | 21.05 | 19.01 | 23.08 |
| Noncore | 22.52 | 20.56 | 24.49 |
| **Median HH Income** |  |  |  |
| Quartile 1, 0-25th percentile | 50.04 | 45.83 | 54.25 |
| Quartile 2, 26-50th percentile | 20.87 | 19.52 | 22.23 |
| Quartile 3, 51-75th percentile | 13.68 | 12.79 | 14.58 |
| Quartile 4, 76-100th percentile | 7.09 | 6.52 | 7.66 |

ZIP code income quartiles for 2016: Quartile 1: 1-42,999; Quartile 2: 43,000-53,999; Quartile 3: 54,000-70,999; Quartile 4: 71,000+. ZIP code income quartiles for 2017: Quartile 1: 1-43,999; Quartile 2: 44,000-55,999; Quartile 3: 56,000-73,999; Quartile 4: 74,000+. ZIP code income quartiles for 2018: Quartile 1: 1-45,999; Quartile 2: 46,000-58,999; Quartile 3: 59,000-78,999; Quartile 4: 79,000+.

### Supplemental Exhibit 4. Nonfatal Firearm Injury Episodes, Patient Characteristics, Rate per 100,000 with 95% Confidence Intervals, 2016 to 2018

| **Category** | **Total (n=228,380)** | **Lower Estimate**  **(n=213,824)** | **Upper Estimate**  **(n=242,936)** |
| --- | --- | --- | --- |
| **Total Nonfatal Firearm Injury Episodes** | 23.40 | 21.91 | 24.89 |
| **Sex** |  |  |  |
| Male | 41.47 | 38.83 | 44.12 |
| Female | 5.84 | 5.43 | 6.24 |
| **5-year Age Group** |  |  |  |
| 0 to 4 | 1.40 | 1.14 | 1.65 |
| 5 to 9 | 1.97 | 1.68 | 2.27 |
| 10 to 14 | 5.71 | 5.07 | 6.34 |
| 15 to 19 | 55.84 | 51.77 | 59.91 |
| 20 to 24 | 73.53 | 67.86 | 79.20 |
| 25 to 29 | 59.08 | 54.86 | 63.30 |
| 30 to 34 | 42.01 | 39.06 | 44.96 |
| 35 to 39 | 30.72 | 28.56 | 32.89 |
| 40 to 44 | 22.00 | 20.37 | 23.63 |
| 45 to 49 | 17.24 | 16.05 | 18.44 |
| 50 to 54 | 13.88 | 12.92 | 14.83 |
| 55 to 59 | 9.20 | 8.45 | 9.96 |
| 60 to 64 | 6.92 | 6.35 | 7.49 |
| 65 to 69 | 5.91 | 5.40 | 6.42 |
| 70 to 74 | 5.02 | 4.49 | 5.55 |
| 75 to 79 | 4.03 | 3.48 | 4.59 |
| 80 to 84 | 3.48 | 2.84 | 4.12 |
| 85+ | 3.19 | 2.30 | 4.08 |
| **Race (Inpatient admissions only)** |  |  |  |
| White | 3.95 | 3.71 | 4.19 |
| Black | 36.82 | 33.22 | 40.41 |
| Hispanic | 7.35 | 6.62 | 8.07 |
| Asian or Pacific Islander | 1.43 | 1.17 | 1.69 |
| Native American | 8.67 | 6.71 | 10.63 |
| Other | 14.41 | 12.04 | 16.78 |
